# Trichloroethylene exposure and somatic mutations of the *VHL *gene in patients with Renal Cell Carcinoma

**DOI:** 10.1186/1745-6673-2-13

**Published:** 2007-11-12

**Authors:** Barbara Charbotel, Sophie Gad, Delphine Caïola, Christophe Béroud, Joelle Fevotte, Alain Bergeret, Sophie Ferlicot, Stéphane Richard

**Affiliations:** 1 UMRESTTE, Université Lyon 1, Université de Lyon, Domaine Rockefeller, Lyon, F-69373, France; 2 Hospices Civils de Lyon, Service des maladies professionnelles, Centre Hospitalier Lyon Sud, F-69495 Pierre Bénite, France; 3 Laboratoire de Génétique Oncologique EPHE, Faculté de Médecine Paris-Sud, Le Kremlin-Bicêtre 94275 Le Kremlin-Bicêtre Cedex, France; 4 CNRS FRE-2939, Institut de Cancérologie Gustave Roussy (IGR), 94805 Villejuif, France; 5 Laboratoire de Génétique Moléculaire, CHU de Montpellier, Institut Universitaire de Recherche Clinique (IURC), INSERM, U 827, Montpellier, F-34000 France; 6 Laboratoire d'Anatomie Pathologique, CHU de Bicêtre, 94275 Le Kremlin-Bicêtre Cedex, France

## Abstract

**Background:**

We investigated the association between exposure to trichloroethylene (TCE) and mutations in the von Hippel-Lindau *(VHL) *gene and the subsequent risk for renal cell carcinoma (RCC).

**Methods:**

Cases were recruited from a case-control study previously carried out in France that suggested an association between exposures to high levels of TCE and increased risk of RCC. From 87 cases of RCC recruited for the epidemiological study, 69 were included in the present study. All samples were evaluated by a pathologist in order to identify the histological subtype and then be able to focus on clear cell RCC. The majority of the tumour samples were fixed either in formalin or Bouin's solutions. The majority of the tumours were of the clear cell RCC subtype (48 including 2 cystic RCC). Mutation screening of the 3 *VHL *coding exons was carried out. A descriptive analysis was performed to compare exposed and non exposed cases of clear cell RCC in terms of prevalence of mutations in both groups.

**Results:**

In the 48 cases of RCC, four *VHL *mutations were detected: within exon 1 (c.332G>A, p.Ser111Asn), at the exon 2 splice site (c.463+1G>C and c.463+2T>C) and within exon 3 (c.506T>C, p.Leu169Pro).

No difference was observed regarding the frequency of mutations in exposed versus unexposed groups: among the clear cell RCC, 25 had been exposed to TCE and 23 had no history of occupational exposure to TCE. Two patients with a mutation were identified in each group.

**Conclusion:**

This study does not confirm the association between the number and type of *VHL *gene mutations and exposure to TCE previously described.

## Background

Renal cell carcinoma (RCC), the most frequent malignancy in the adult kidney, is usually sporadic [1]. The phenotype is extremely heterogeneous and several classifications of renal epithelial tumours have been proposed [2,3]. Main histological subtypes of renal epithelial tumours include clear-cell RCC (75%), papillary RCC (10–15%), chromophobe RCC (5%) and oncocytomas (5%). Inactivation of the *VHL *tumour suppressor gene is thought to result both in development of tumors in the von Hippel-Lindau (VHL) disease (MIM #19330) and in sporadic clear-cell RCC [4]. Germline mutations of *VHL *gene are responsible for VHL disease, a rare dominantly inherited cancer syndrome predisposing to a number of highly vascularized tumors including multiple clear-cell RCC whereas somatic mutation or methylation of *VHL *gene is a frequent event in sporadic clear-cell RCC [4,5]. Trichloroethylene (TCE) is a solvent used in numerous industries, as degreaser in the metal manufacturing industry, solvent for oils and resins, or the production of rubber and as a chemical intermediate in the production of refrigerants. A number of epidemiological studies have investigated the association between exposure to trichloroethylene and renal cell cancer but the results have been inconsistent [6]. A cohort study conducted in a cardboard factory and a case-control study conducted in the same area in Germany found significantly elevated risk for renal cell cancer and trichloroethylene occupational exposure [7,8]. For subjects in that case control study, a specific pattern of mutations in the *VHL *gene has been reported in RCC cases with former prolonged and high-level exposures to trichloroethylene [9]. However, another study carried out in Germany to evaluate the phenotype and the genotype of renal tumours in occupationally TCE-exposed patients revealed no unique phenotype, genotype or mutation pattern in the *VHL *gene of renal tumours after TCE exposure [10].

In France, the results of a case-control study performed in a geographic area with a high frequency and intensity of exposure suggested an association between exposures to high levels of TCE and increased risk of RCC [6,11,12]. Thus it seemed to be of interest to further expand this case-control study with a molecular analysis to confirm or not the specific pattern of *VHL *mutations associated with trichloroethylene exposure reported in the German study. The objective of the study was to test the hypothesis of an association between exposure to trichloroethylene and *VHL *mutations and the subsequent risk for RCC.

## Materials and Methods

### Tumour samples

Cases were recruited from the case-control study previously carried out in the Arve Valley [6,11,12]. For the specific analysis on *VHL *mutations, patients were informed of the objectives of this study and a new written consent was requested. With the patients' consent (or that of their next-of-kin) we collected tumour tissues from the various pathologists. General information and exposure data were obtained from the epidemiological study.

A total of 87 cases of renal cell cancer had been recruited for the epidemiological study. From these, 69 accepted to be included in the present study. Formalin and Bouin's fixed paraffin-embedded tissue samples from 64 patients with renal tumours were cut and transferred onto glass slides. Furthermore, 5 frozen tumours were included in this series. After H&E staining, all samples were evaluated by a specialized pathologist in order to identify the histological subtype and then focus on clear cell RCC. Forty-one samples were paraffin-embedded tissue fixed in Bouin's and 23 in formalin solution. The majority of the tumours were of the clear cell RCC subtype: 46 were solid clear cell RCC and 2 were cystic clear cell RCC.

### DNA extraction

Genomic DNA was extracted from tumour samples with ten 7 μm sections of paraffin blocks loaded on standard slides focusing on the 48 clear cell RCC previously identified. The blade was cleaned after each block to prevent cross contamination between the samples. Each slide was scraped with a scalpel in order to recover only tumour tissue, by comparison with an H&E staining of the same section. The QIAamp DNA Mini Kit (Qiagen, Courtaboeuf, France) was used, with 2 slight modifications. First, samples were digested during 18 hours at 56°C with proteinase K to obtain complete digestion. Second, paraffin was not removed by xylene extraction: we used instead the fact that the paraffin wax melts during the later 56°C incubation. Microtubes were centrifuged at full speed for 10 min at 4°C to remove all paraffin. Finally, the total amount of DNA was measured by Nanodrop technology (Labtech France, Paris, France).

### Mutation analysis

Mutation screening of the 3 *VHL *coding exons and exon-intron junctions was performed thanks to two groups of primers available on request. The first group of primers was designed to amplify exon 1 in two overlapping fragments, exon 2 and exon 3 in one fragment each, the four fragments comprising approximately 300 bp. The second group was designed to amplify exon 1 in 4 fragments, exons 2 and 3 in two fragments each, corresponding to fragments of 100–150 bp. Since the yield after PCR amplification was not always satisfactory, nested PCR were performed with 25 and then 35 cycles. Briefly, tumour DNA aliquots were used in a 10 μL final reaction volume comprising 1X Q solution and buffer with 15 mM of MgCl2, 0.8 mM of dNTP mix, 0.3 μM of each forward and reverse primers and 0.5 U of HotStarTaq^® ^DNA Polymerase (Qiagen). A negative control was introduced in all PCR experiments. PCR products were analyzed on standard 1.5% agarose gels stained with ethidium bromide (0.5μg/mL) before purification with ExoSAP-IT (Amersham Biosciences, Saclay, France). Sequencing reactions were performed using Big Dye Terminator (Applied Biosystems, Courtaboeuf, France), purified through Sephadex G-50 (Amersham Biosciences) and run on an ABI 3730 Genetic Analyzer (Applied Biosystems). Sequence files were aligned and analyzed by Sequencher v4.2.2 (Gene Codes Corporation, Ann Arbor, USA) software. All sequence alterations were verified independently by reamplifying the corresponding fragment and repeating the sequencing procedure using both forward and reverse primers.

### Statistical analysis

The mutation data were analyzed with the UMD-*VHL *software [13] to compare them with previous somatic mutations [25].

A descriptive analysis was performed to compare exposed and non exposed cases of clear cell RCC in terms of prevalence of mutations in both groups.

The statistical analysis was performed using the SAS system 9.1.3.

### Legal agreement

Before starting the study an agreement was obtained from the CCPPRB Lyon A (consultative committee for persons protection in biomedical research). Then an approval was obtained from the French Ministry of Research (*Comité consultatif pour le traitement de l'information en matière de recherche dans le domaine de la santé*) and the French data protection authority (*Commission Nationale de l'Informatique et des Libertés*) was informed about the study.

## Results

### Mutation analysis

**Figure 1:**
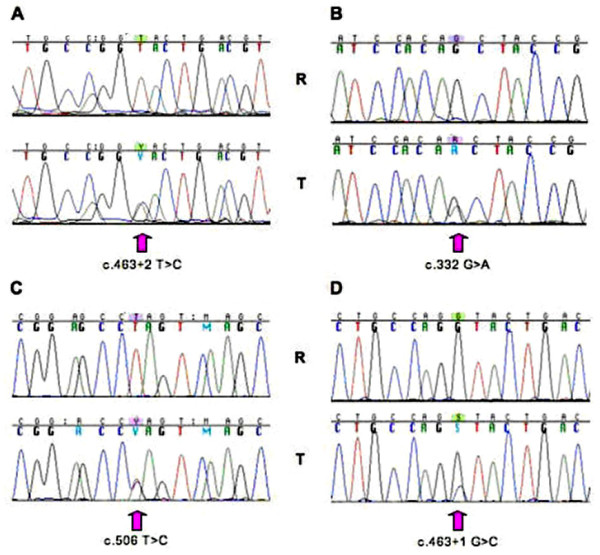
Sequence chromatograms of the 4 mutations identified in the *VHL *gene in this study. R and T are DNA from a commercially available reference and tumour tissue tested respectively. Panels A, B and C correspond to 3 different renal clear cell tumours fixed in formalin solution, whereas panel D corresponds to one of the frozen tumours.

Mutation screening was performed on the 48 confirmed cases of clear cell RCC, comprising 26 Bouin's fixed tumours, 17 were formalin fixed and 5 frozen tumours. For all of the frozen samples the *VHL *gene was successfully PCR amplified and sequenced, compared to 71% (12 of 17) of the formalin-fixed samples, and 38% (10 of 26) of the Bouin's fixed tissues. Thus, the *VHL *gene was entirely sequenced (ie 100% of the coding sequence analysed) for a total of 26 tumours (54%). A *VHL *mutation was detected in one of the frozen samples at the exon 2 splice site (c.463+1G>C). Furthermore, three *VHL *mutations were detected in the fixed tumours: one mutation within exon 1 (c.332G>A, p.Ser111Asn), one at the exon 2 splice site (c.463+2T>C), and the last one within exon 3 (c.506T>C, p.Leu169Pro), see Figure 1. These three cases were fixed in formalin solution. No mutations were found in the samples fixed in Bouin's solution. Regarding the codon 81, 75% of the samples (31 fixed and 5 frozen tumours respectively) were successfully sequenced for the corresponding PCR fragment in exon 1 and no mutation at this particular codon was observed.

### Exposure to TCE

The mean of sequencing percentage was 86.1 (+/-19.5) among exposed cases and 80.3 (+/-27.3) among non-exposed cases, this difference is not significant (p = 0.40). The sequencing rate reached 100% for 15 cases (60.0%) of the exposed group versus 11 (48%) cases of the non exposed group (p = 0.30).

The codon 81 was seen for 20 (80%) of the exposed cases and 16 (70%) of the non exposed cases (p = 0.40).

No difference was observed regarding the frequency of mutations in exposed versus unexposed groups (Table 1). Indeed, among the clear cell RCC, 25 had been exposed to TCE and 23 had no history of occupational exposure to TCE. Two patients with a mutation were identified in each group. In the exposed group, 9 (36%) patients had been exposed to a low cumulative dose, 4 (16%) to a medium cumulative dose and 12 (48%) to a high cumulative dose of TCE.

**Table 1:** Mutation frequency according to exposure to TCE

		**No mutation**	**Mutation**	**Fisher bilateral exact test (p)**
**Sequencing percentage: any rate** **N = 48**	Non exposed to TCE N = 23	21 (91%)	2 (9%)	1.00
	Exposed to TCE N = 25	23 (92%)	2 (8%)	
**Sequencing percentage 100%** **N = 26**	Non exposed to TCE N = 11	9 (82%)	2 (18%)	1.00
	Exposed to TCE N = 15	13 (87%)	2 (13%)	

If we consider only renal clear cell tumours for which the *VHL *gene was entirely sequenced, among 15 patients who had been exposed to TCE, 6 (40%) had been exposed to a low cumulative dose, 3 (20%) to a medium cumulative dose and 6 (40%) to a high cumulative dose. Two of the mutations identified occurred in TCE exposed cases but only one of these had been highly exposed. Description of exposures in patients for which a mutation was identified is presented in Table 2.

**Table 2:** Description of patient with a *VHL *gene mutation

	**Patient A**	**Patient B**	**Patient C**	**Patient D**
**Type of mutation**	c.463+2 T>C	c.332 G>A	c.506 T>C	c.463+1 G>C
**Age at diagnosis**	52 years	58 years	63 years	42 years
**Sex**	man	women	man	man
**Exposure to TCE**	yes	no	yes	no
**Conditions of exposure**	2 years to 15 ppm in screw cutting industry during		highly exposed (cumulative dose 830 ppm.years with peaks)	
**Other occupational exposures identified**	cutting oils asbestos	cutting oils in screw cutting industry	other chlorinated solvents, cutting oils, lead, ionizing radiations, asbestos and welding fumes	none of the exposures studied
**Tobacco smoking**	12 pack-years	never	20 pack-years	never
**Body Mass Index**	< 25	> 30	< 25	between 25 and 30

## Discussion

It has been postulated that sporadic and familial renal cell carcinomas have a common carcinogenic pathway and that at least one gene should be altered in both forms. This has been confirmed with the cloning of the *VHL *gene and the identification of germline and somatic mutations of this gene in VHL patients and sporadic RCC. Today, more than 400 germline mutations have been reported in VHL patients and about 300 somatic mutations in sporadic RCC [13]. The collection of a large number of mutations is necessary to identify key residues in the biological function of the protein, for molecular epidemiology and to establish correlations between the localization of the mutation and specific phenotypes. In a previous study, Beroud et al. [14-16] have shown that the somatic and germline mutational events are different with only 22% of missense mutations in sporadic RCC vs 63% in familial cases (p < 0.001). They also showed that the distribution of missense mutations is different in the two groups with 68% of missense mutations corresponding to transversion in somatic mutations vs 35% in germline mutations (p < 0.001). Since it is thought that transversions implicate an extrinsic factor, these data support the hypothesis of the involvement of environmental factors in the aetiology of sporadic RCC [17-19]. Brüning et al. studied the RCC incidence in individuals with former prolonged and high-level exposures to TCE [20]. They found a specific pattern of *VHL *somatic mutations with a high frequency in exon 2. In 1999, Brauch et al. completed these data and showed multiple intragenic mutations within the same tumour (42% of cases), a very high proportion of C>T transitions (70%) and a hot spot of mutations at codon 81 (p.Pro81Ser) [9]. Altogether these data suggest a role of TCE or its metabolites in these mutational events. To explore this further, we have compared the distribution of somatic *VHL *mutations in RCC from patients exposed to TCE (from Brüning [20] and Brauch [9]) with the RCC somatic mutations in the UMD-*VHL *database [13] that collects all published mutations of the *VHL *gene. The purpose of this analysis was to compare specific pattern of *VHL *mutations thought to be associated with TCE exposure with a wider collection of *VHL *gene information.

This comparison confirms that the specific pattern of mutations identified by Brüning [20] and Brauch [9] is different from the pattern in the large UMD-*VHL *database of somatic *VHL *mutations in RCC tumours. In fact, this somatic mutation pattern suspected to be associated with TCE exposures is much closer to the germline mutation profile with an excess of missense mutations. In addition, 50% (16 out of 32) of these missense mutations are C>T transitions with 13 involving codon 81. As these transitions do not involve a CpG dinucleotide, we can suspect that they result from the exposure to a toxic substance or any other carcinogen.

In addition the distribution of somatic mutations reveals that the codon 81 has been involved in only one tumour from studies other than those of Brüning et al [20] and Brauch et al. [9]. Overall mutations at this position could be specifically associated to exposure to trichloroethylene. It is however surprising, considering the intensive use of TCE all over the world, that this specific mutation has not been reported in other case series. In our series, no mutation was detected at this particular codon, indicating that, as observed by Schraml et al [10], we have not confirmed the results of Brüning et al. [20] and Brauch et al. [9].

The present study was performed in blinded test fashion regarding TCE exposure data. The series comprised 48 cases of confirmed clear cell RCC. All of these cases had been included in a case control study previously published. 26 of the tumours were Bouin's solution-fixed, 17 were formalin-fixed and 5 were frozen tumours. As reported in the literature, molecular analysis based on archival tissues is possible however often difficult [21]. Actually, formic acid contained in formalin solution, fixation time and period of storage of the tissue blocks often affect the quality of DNA. Furthermore, picric acid contained in Bouin's solution is known to degrade nucleic acids, thus a low yield of PCR amplification of DNA could be obtained. Here 26 tumours (21 fixed and 5 frozen tumours, ie 54%) were successfully analyzed regarding the *VHL *gene. However, codon 81 was seen for 80% of the cases of clear cell RCC who had been exposed to TCE. Three mutations were detected in the fixed tumours, within exon 1 (c.332G>A, p.Ser111Asn), exon 2 splice site (c.463+2T>C) and exon 3 (c.506T>C, p.Leu169Pro). These three cases were fixed in formalin solution. No mutation was found in the samples fixed in Bouin's solution. Regarding the 5 frozen tumours, one mutation was detected at the exon 2 splice site (c.463+1G>C). The 4 mutations detected in the present study have been described in previous *VHL *studies: they are reported between 1 and 4 times either at the germline or somatic level in the *VHL *Mutation Database [25].

Interestingly, it is reported in the literature that a *VHL *somatic mutation is detected in approximately half of sporadic clear cell RCC [14,22,23]. Thus, among the 48 clear cell RCC tumours analyzed in the present study, at least 20 samples would be expected to carry a *VHL *mutation compared to 4 mutations detected here. However, these 4 mutations were detected in a series of 26 clear cell RCC for which the *VHL *gene was entirely sequenced, corresponding to 15%. As a number of fixed samples could not be successfully PCR amplified because of DNA degradation, we cannot exclude that some of these samples carry a *VHL *mutation. However, it was recently suggested a new cause of occupational cancer where there was a molecular analysis of *VHL *without mutation detected in this gene, suggesting that other genes may be implicated in RCC and in particular linked to chemical exposure [24].

The *VHL *gene can be inactivated somatically, besides loss of heterozygosity, either by mutation or promoter hypermethylation. The quality of the DNA extracted from fixed tumours did not allow us to analyze the methylation status of the *VHL *promoter. Thus, we cannot exclude that some tumours may involve hypermethylation.

In the present study, 25 (52%) of the clear cell RCC cases concerned patients who had been exposed to TCE, 12 (48%) of them had been exposed to high cumulative dose. Despite this high rate of exposure to TCE and the rate of complete sequencing (100% for 26 of 48 cases of RCC), only two of the patients for which a mutation was identified had been exposed to TCE and only one of them had been highly exposed the cumulative exposure reaching 830 ppm× years. However this patient had also been exposed to many other occupational risks including exposures to carcinogens (asbestos and ionizing radiation). Three of the patient for which a mutation was identified had been exposed to cutting oils.

When considering cases of renal clear cell cancers with a rate of *VHL *sequencing reaching 100%, the rate of mutations was not statistically different among patients who had been exposed to TCE and among those who had not, respectively 13% and 18%.

In summary we conclude that this study has not confirmed the association between the number and type of *VHL *gene mutations and exposure to TCE previously described. However, due to methodological limitations, these results do not allow to totally rule out this specific association.

## Competing interests

The author(s) declare that they have no competing interests.

## Authors' contributions

BC and CB studied published data and conceived the study. SG, DC, SF and SR performed the pathology and DNA analysis. JF performed the exposure assessment. AB was scientific manager of the epidemiological study previously published and contributed to the conception of the present study.

BC performed the statistical analyses and wrote the final version of the paper with SG and SR. All authors read and approved the manuscript.

## References

[B1] Cohen HT, McGovern FJ (2005). Renal-cell carcinoma. N Engl J Med.

[B2] Thoenes W, Störkel S, Rumpelt J (1986). Histopathology and classification of renal cell tumors. Path Res Pract.

[B3] Kovacs G, Akhtar M, Beckwith BJ, Bugert P, Cooper CS, Delahunt B, Eble JN, Fleming S, Ljungberg B, Medeiros LJ, Moch H, Reuter VE, Ritz E, Roos G, Schmidt D, Srigley JR, Störkel S, van den Berg E, Zbar B (1997). The Heidelberg classification of renal tumors. J Pathol.

[B4] Kaelin WG (2002). Molecular basis of the *VHL *hereditary cancer syndrome. Nat Rev Cancer.

[B5] Richard S, Graff J, Lindau J, Resche F (2004). Von Hippel-Lindau disease. Lancet.

[B6] Charbotel B, Fevotte J, Hours M, Martin JL, Bergeret A (2006). Case-control study on renal cell cancer and occupational exposure to trichloroethylene. Part II: Epidemiological aspects. Ann Occup Hyg.

[B7] Henschler D, Vamvakas S, Lammert M, Dekant W, Kraus B, Thomas B, Ulm K (1995). Increased incidence of renal cell tumors in a cohort of cardboard workers exposed to trichloroethylene. Arch Toxicol.

[B8] Vamvakas S, Brüning T, Thomasson B, Lammert M, Baumüller A, Bolt HM, Dekant W, Birner G, Henschler D, Ulm K (1998). Renal cell cancer correlated with occupational exposure to trichloroethene. J Cancer Res Clin Oncol.

[B9] Brauch H, Weirich G, Hornauer MA, Storkel S, Wohl T, Bruning T (1999). Trichloroethylene exposure and specific somatic mutations in patients with renal cell carcinoma. J Natl Cancer Inst.

[B10] Schraml P, Zhaou M, Richter J, Brüning T, Pommer M, Sauter G, Mihatsch MJ, Moch H (1999). [Analysis of kidney tumors in trichloroethylene exposed workers by comparative genomic hybridization and DNA sequence analysis]. Verh Dtsch Ges Pathol.

[B11] Charbotel B, Fevotte J, Hours M, Martin JL, Bergeret A (2005). Case-control study on renal cell cancer and occupational trichloroethylene exposure in the Arve valley (France). Final Report Inrets, UCBL.

[B12] Fevotte J, Charbotel B, Muller-Beaute P, Martin JL, Hours M, Bergeret A (2006). Case-control study on renal cell cancer and occupational exposure to trichloroethylene. Part I: Exposure assessment. Ann Occup Hyg.

[B13] Béroud C, Joly D, Gallou C, Staroz D, Orfanelli MT, Junien C (1998). Software and database for the analysis of mutations in the *VHL *gene. Nucleic Acids Res.

[B14] Gallou C, Joly D, Méjean A, Staroz F, Martin N, Tarlet G, Orfanelli MT, Bouvier R, Droz D, Chrétien Y, Maréchal JM, Richard S, Junien C, Béroud C (1999). Mutations of the *VHL *gene in sporadic Renal Cell Carcinoma. Definition of a risk factor for *VHL *patients to develop a RCC. Hum Mutat.

[B15] Longuemaux S, Delomenie C, Gallou C, Mejean A, Vincent-Viry M, Bouvier R, Droz D, Krishnamoorthy R, Galteau MM, Junien C, Béroud C, Dupret JM (1999). Candidate genetic modifiers of individual susceptibility to renal cell carcinoma: a study of polymorphic human xenobiotic-metabolizing enzymes. Cancer Res.

[B16] Gallou C, Longuemaux S, Deloménie C, Méjean A, Martin N, Martinet S, Palais G, Bouvier R, Droz D, Krishnamoorthy R, Junien C, Béroud C, Dupret J-M (2001). Association of GSTT1 non-null and NAT1 Slow/Rapid genotypes with *VHL *transversions in sporadic Renal Cell Carcinoma. Pharmacogenetics.

[B17] La Vecchia C, Negri E, D'Avanzo B, Franceschi S (1990). Smoking and renal cell carcinoma. Cancer Res.

[B18] Malker H, Malker B (1984). Kidney cancer among leather workers. Lancet.

[B19] Yu M, Mack T, Hanisch R, Cicioni C, Henderson BE (1986). Cigarette smoking, obesity, diuretic use and coffee consumption as risk factors for renal cell carcinoma. J Natl Cancer Inst.

[B20] Bruning T, Weirich G, Hornauer MA, Hofler H, Brauch H (1997). Renal cell carcinomas in trichloroethene (TRI) exposed persons are associated with somatic mutations in the von Hippel-Lindau (*VHL*) tumour suppressor gene. Arch Toxicol.

[B21] Imyanitov EN, Suspitsin EN, Buslov KG, Kuligina ES, Belogubova EV, Togo AV, Hanson KP, Jan Kieleczawa (2006). Isolation of Nucleic Acids from Archival Tissues and Other Difficult Sources. DNA Sequencing II: Optimizing Preparation and Cleanup.

[B22] Gnarra JR, Tory K, Weng Y, Schmidt L, Wei MH, Li H, Latif F, Liu S, Chen F, Duh FM (1994). Mutations of the *VHL *tumour suppressor gene in renal carcinoma. Nat Genet.

[B23] Kim WY, Kaelin WG (2004). Role of *VHL *gene mutation in human cancer. J Clin Oncol.

[B24] Richard S, Carrette MN, Béroud C, Ferlicot S, Imbernon E, Iwatsubo Y, Egloff H, Sordet D, Salé JM (2004). High incidence of renal tumours in vitamins A and E synthesis workers: a new cause of occupational cancer?. Int J Cancer.

[B25] The VHLmutations database. http://www.umd.be:2020/.

